# Supplementary material for: Tissue-aware interpretation of genetic variants advances the etiology of rare diseases
Source: Mol Syst Biol. 2024 Sep 16;20(11):4. doi: 10.1038/s44320-024-00061-6 (PMC11535248; doi:10.1038/s44320-024-00061-6)
Supplement: Supplementary file 1 — Appendix [file 44320_2024_61_MOESM1_ESM.pdf]

APPENDIX: Tissue-aware interpretation of genetic variants advances the etiological understanding of rare diseases

Chanan M. Argov, Ariel Shneyour, Juman Jubran, Eric Sabag, Avigdor Mansbach, Yair Sepunaru, Emmi Filtzer, Gil Gruber, Miri Volozhinsky, Yuval Yogev, Ohad Birk, Vered Chalifa-Caspi, Lior Rokach, Esti Yeger-Lotem

Contents

This file contains Appendix Figures S1-9 and Appendix Tables S1-4

Appendix Figure S1 ………….. 2

Appendix Figure S2 ………….. 3

Appendix Figure S3 ………….. 5

Appendix Figure S4 ………….. 6

Appendix Figure S5 ………….. 7

Appendix Figure S6 ………….. 8

Appendix Figure S7 ………….. 9

Appendix Figure S8 ………….. 10

Appendix Figure S9 ………….. 11

Appendix Table S1 ………….. 12

Appendix Table S2 ………….. 14

Appendix Table S3 ………….. 17

Appendix Table S4 ………….. 23

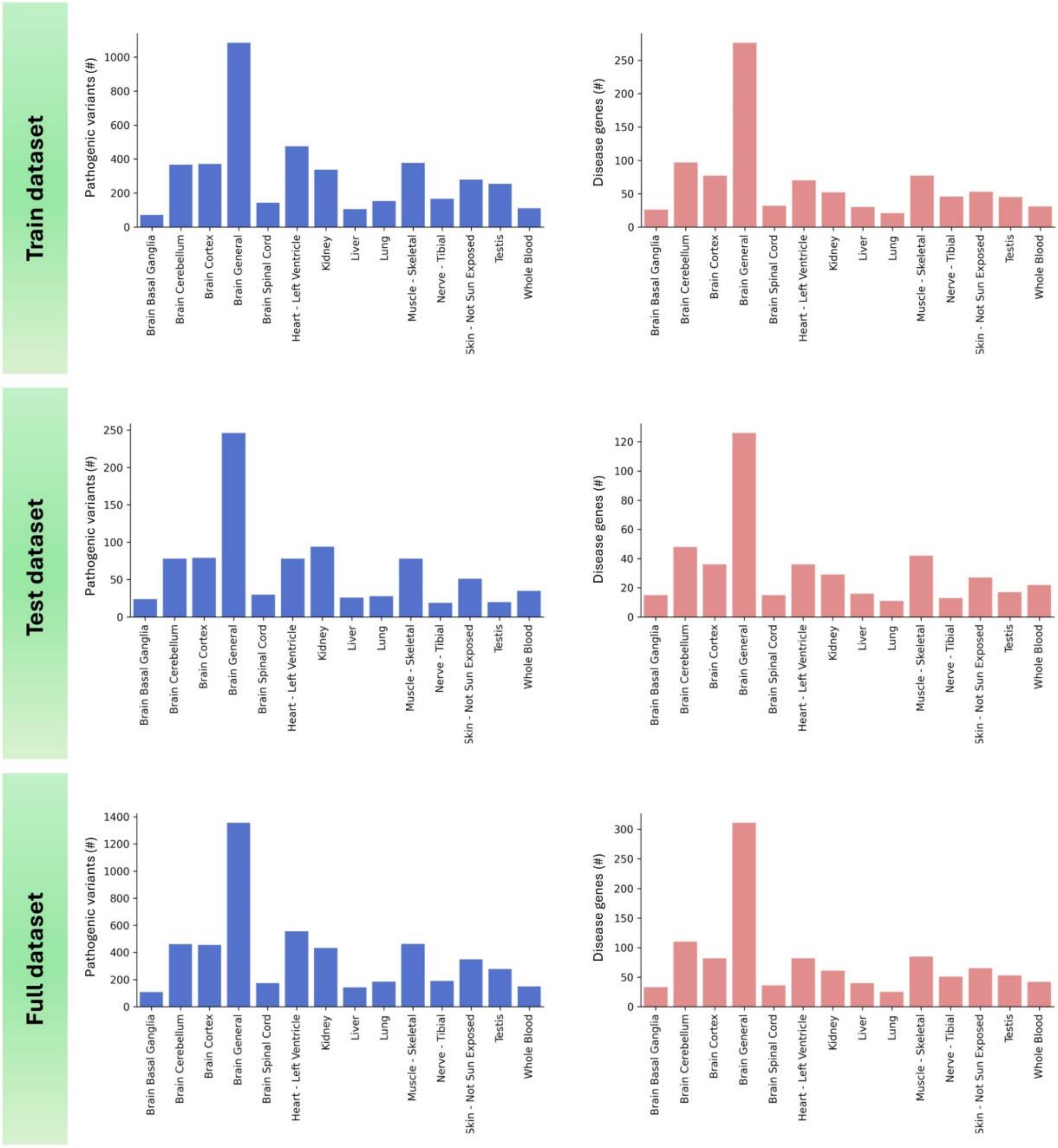

Appendix Fig S1. The number of pathogenic variants and the number of respective disease genes per tissue in the different data sets.

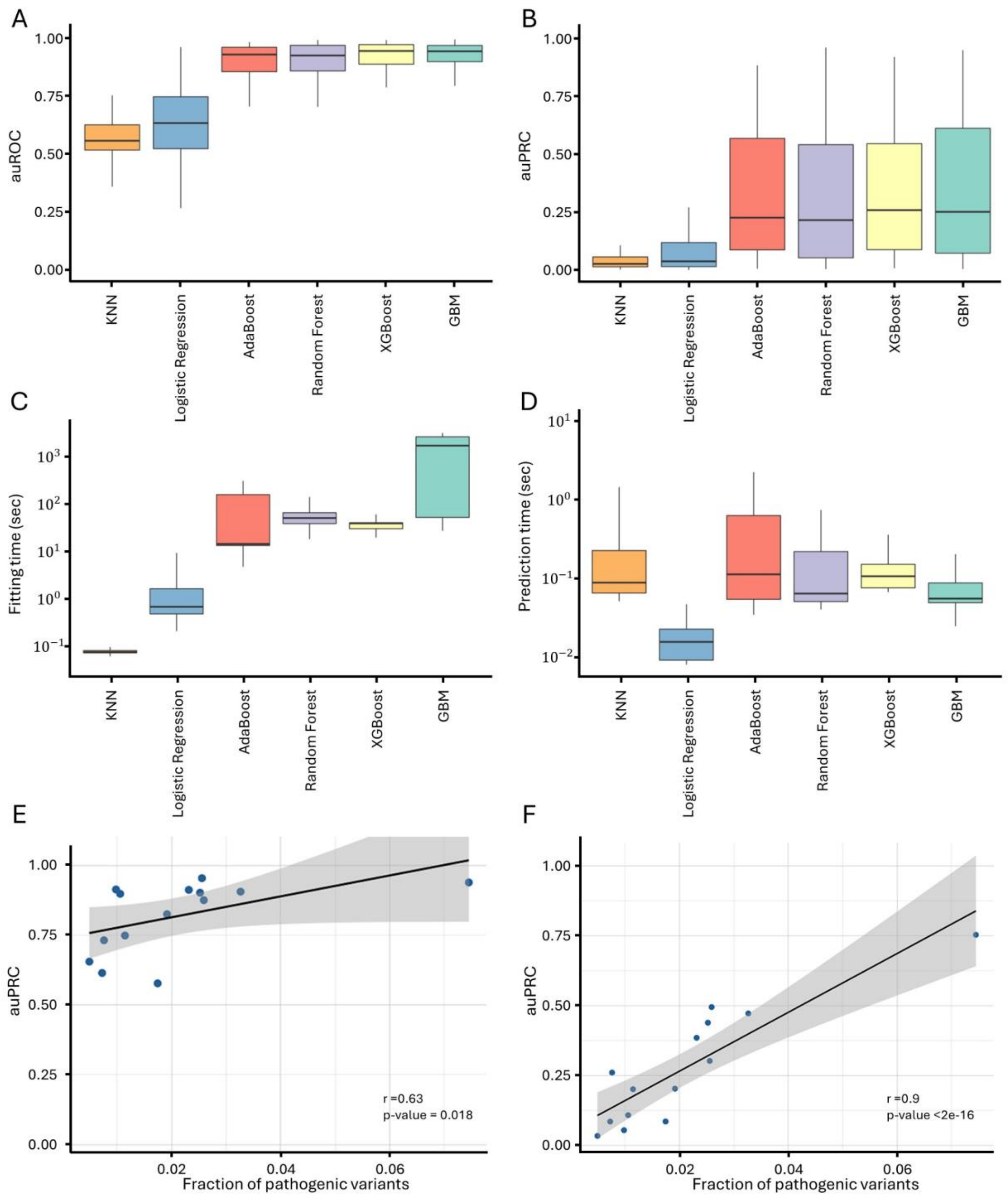

A
auROC
B
auPRC
KNN
Logistic Regression
AdaBoost
Random Forest
XGBoost
GBM
C
Fitting time (sec)
D
Prediction time (sec)
E
F
auPRC
r =0.63
p-value = 0.018
r =0.9
p-value <2e-16
Fraction of pathogenic variants

Appendix Fig. S2. Assessment of TRACEvar implementations.

A. The mean auROC of six machine-learning methods in 14 tissue models. Performance was measured via 10-fold cross validation over the training set. Gradient boosting machine (GBM) and XGBoost had significantly higher auROC than other methods (adjusted p=0.0009 and 0.0004, respectively, one tail paired Wilcoxon test).

B. The mean auPRC of the same methods, measured as described in A. GBM and XGBoost had significantly higher auPRC than other methods (adjusted p=0.009 and 0.02, one tail paired Wilcoxon test). Since GBM performed best and we favored precision it was selected for TRACEvar implementation.

C. The mean fitting time per method in 14 tissue models.

D. The mean prediction time per method in 14 tissue models

E-F. The Spearman correlation between the fraction of pathogenic variants per tissue in the training set and the auPRC of TRACEvar tissue models. The auPRC were computed for the training set (E, Spearman r=0.63 and p=0.018) and the test set (F, Spearman r=0.9 and p=<2e-16).

SVM=support vector machine; KNN=K-nearest neighbors; LR=logistic regression; RF=random forest.

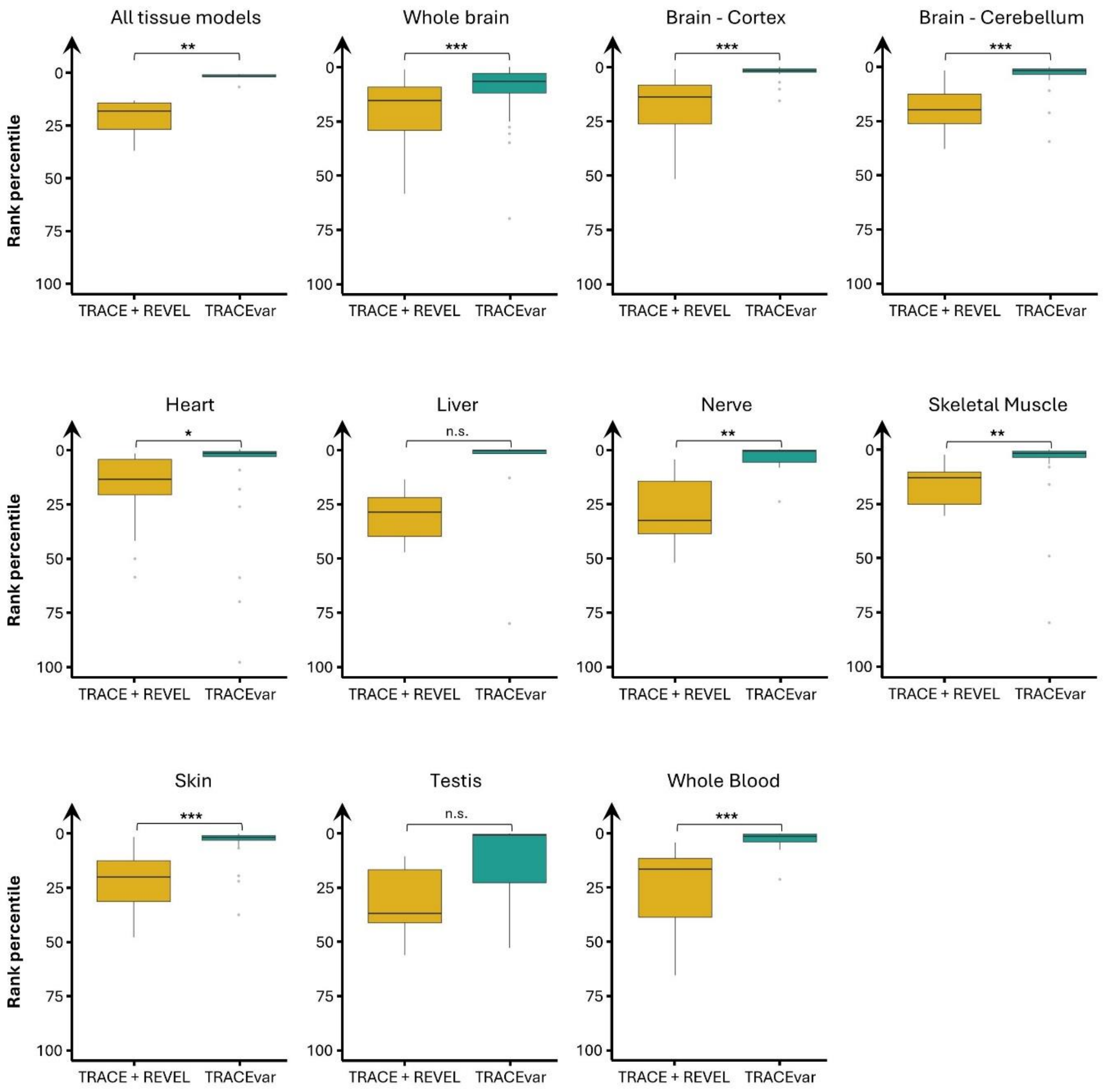

Appendix Fig. S3. Comparison between the performance of TRACEvar and a hybrid approach that combines the tissue-unaware REVEL tool and the tissue-aware TRACE method. TRACEvar outperformed the hybrid approach and was significantly better in 8/10 tissue contexts for which TRACE data were available, and when analyzed collectively (all tissue models). Adjusted p-values:(*) $p<0.05$, (**) $p<0.01$, (***) $p<0.001$, (n.s.) not significant.

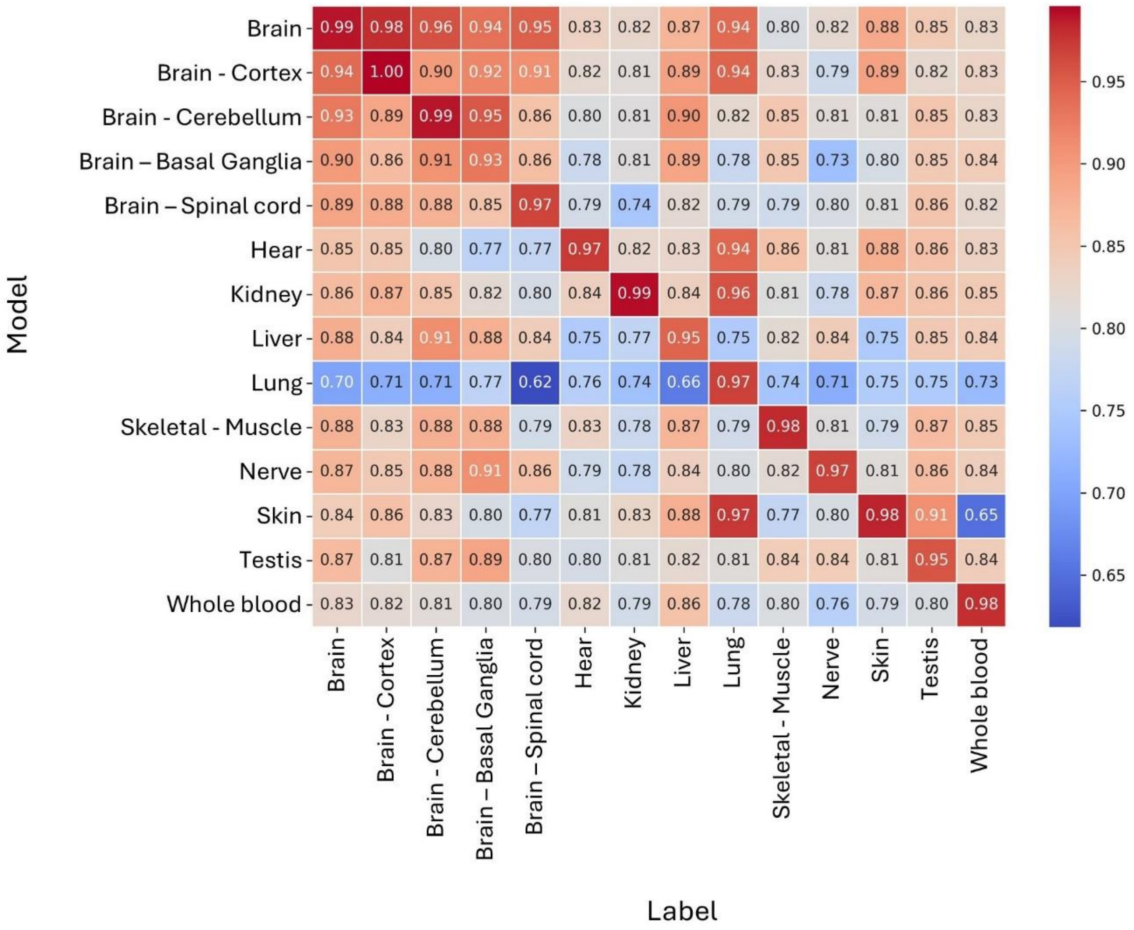

Appendix Fig. S4. The impact of using the wrong TRACEvar tissue model on models' performance. Variants from each tissue (column) were predicted using TRACEvar tissue models (rows), and the heatmap shows the model's performance measured using auROC. The auROC value of the correct tissue model was always the highest, except for lung, which had equally high auROC in the lung and skin models.

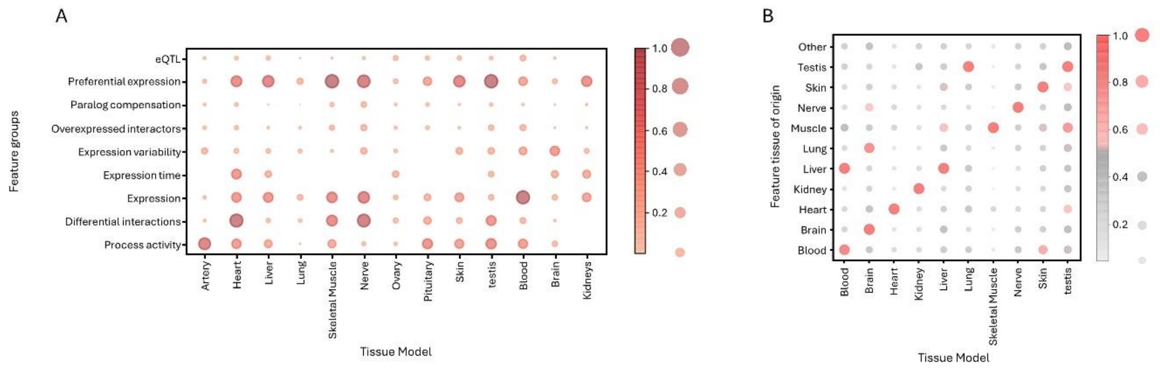

Appendix Fig. S5. The contribution of different types of tissue-specific features to each tissue model. Color scale and dot size reflect the normalized importance values. Models of brain sub-regions were not included due to their similarity.

A. Features were grouped by their type.

B. Features were grouped by their tissue-of-origin (y axis). Features of the modeled tissue were typically more important than features of other tissues

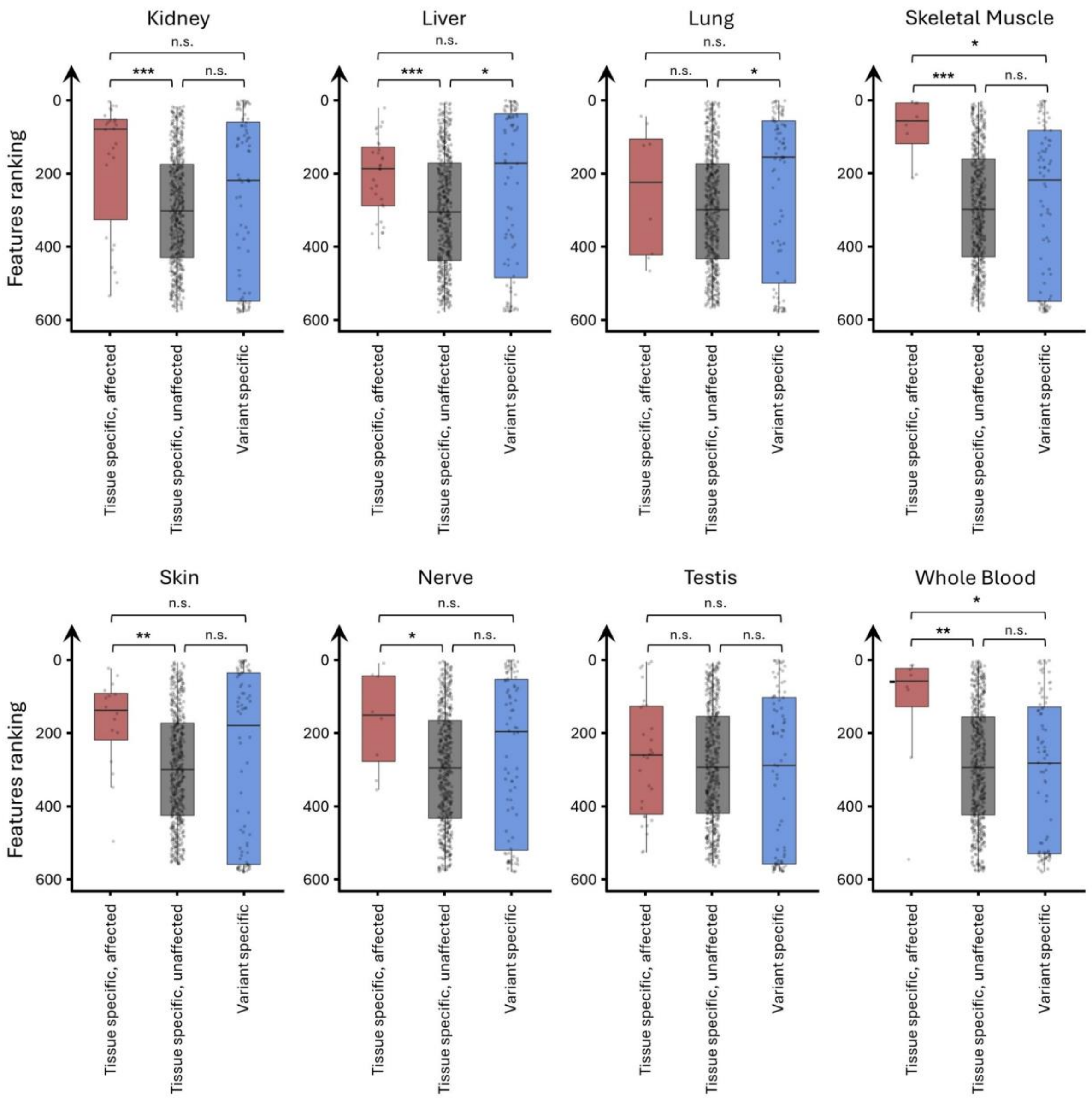

Appendix Fig. S6. The ranking of variant-specific features (blue), tissue-specific features of the modeled tissue (red), and tissue-specific features of other tissues (gray) per tissue model. Comparison was done using MW test. (*) $p<0.05$, (**) $p<0.01$, (***) $p<0.001$, (n.s.) not significant.

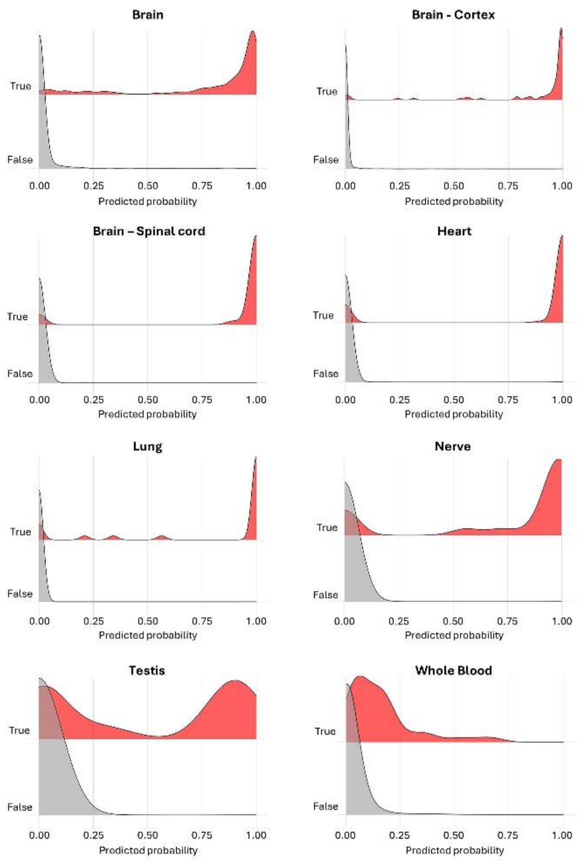

Appendix Fig. S7. The distribution of TRACEvar scores per tissue model for variants in the test set.

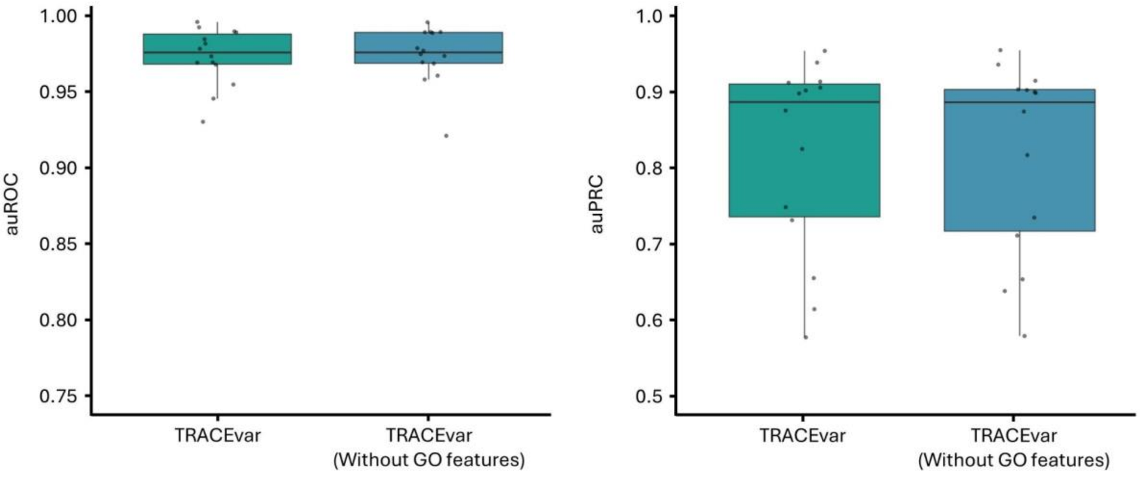

Appendix Fig. S8. The performance of TRACEvar tissue models with and without biological process features. Models were trained on the training dataset and tested on the test dataset. The removal of biological process features had weak effect of TRACEvar performance.

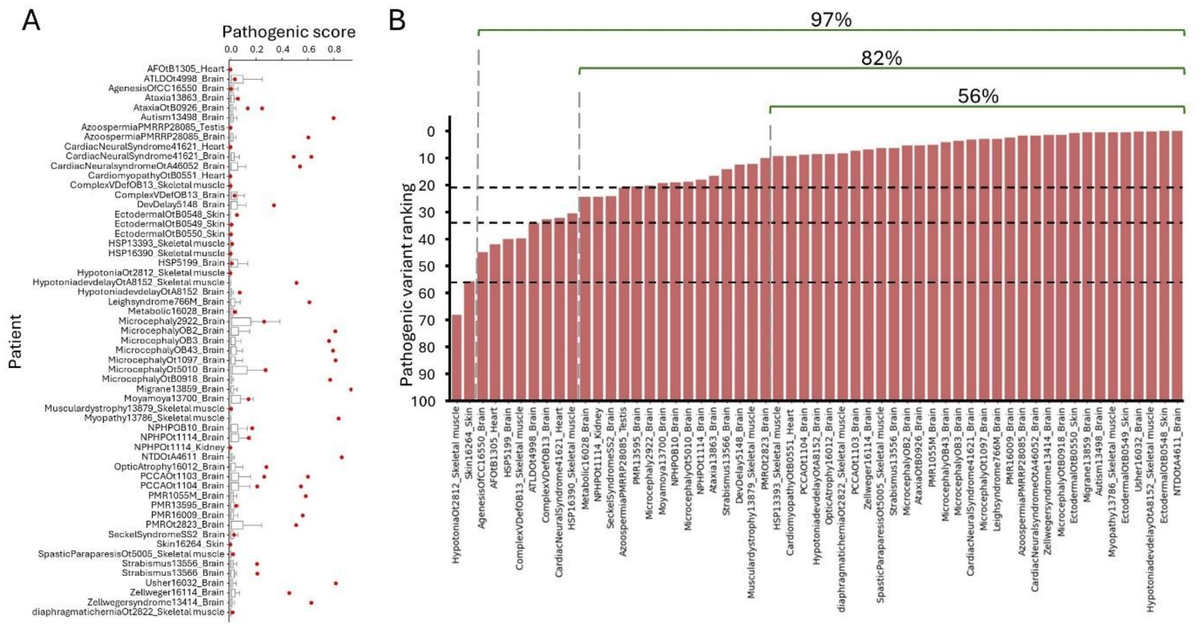

Appendix Fig. S9. Assessment of TRACEvar performance on clinical cases using patient-specific models. Patient-specific models were created upon removing variants in the same genes as the candidate variants of that patient from the training set prior to training.

A. Box plot summaries of TRACEvar pathogenic scores of variants (x axis) in 57 patients (y axis). Each box represents the scores of all candidate variants of one patient case, calculated by the model of the patient's affected tissue. Red dots mark the verified pathogenic variant (one or two per case).

B. The rank of the verified pathogenic variant out of the patient's candidate variants. 97% of the verified pathogenic variants ranked above the median; 82% ranked at the top quartile; 56% ranked at the top 10%.

Table S1. A description of TRACEvar features.

Table S1A. A description of TRACEvar tissue-specific features.

Feature Group	Feature	Number of Features	Description	Links
Expression	Expression	54	Gene expression level in adult tissues	GTEx Portal
	Preferential Expression	54	Preferential expression of the gene in adult tissue samples	TRACE
	Expression Variability	19	Gene expression variability between individuals in adult tissues	TRACE
Expression time	Antenatal to adulthood	134	Gene expression level in newborn to adult tissues	Evo-devo
	Expression Variability (time)	7	Time points specificity of gene expression level across age	TRACE
PPIs	Differential PPIs	44	Differential protein-protein interactions (PPIs) scores of the gene in adult tissues	DiffNet
	Elevated PPIs	54	The number of elevated PPIs of the gene in adult tissues	myProteinNet
Paralogs	Paralog Relationships	53	The ratio between the expression levels of the gene and its paralog in adult tissues	Jubran et al.
eQTL	eQTL	48	eQTL involving the gene in adult tissues	GTEx Portal
Process	Process Activity	28	Activity of GO processes that include the gene in adult tissues	ProAct

Table S1B. A description of variant-specific features from CADD.

Feature Group	Number of Features	Feature Name[1]
Conservation, Evolution	11	priPhCons, mamPhCons, verPhCons, priPhyloP, mamPhyloP, verPhyloP, GerpRS, GerpRSpval, GerpN, GerpS, Grantham
Variation Frequency	10	Freq100bp, Rare100bp, Sngl100bp, Freq1000bp, Rare1000bp, Sngl1000bp, Freq10000bp, Rare10000bp, Sngl10000bp, Dist2Mutation
Variation Consequence	8	Consequence_
Pathogenicity Scores	5	RawScore, PHRED, SIFTval, PolyPhenVal, ConsScore
micro-RNA	3	mirSVR-Score, mirSVR-E, mirSVR-Aln
Domain	5	Domain_
Variation Type	4	Length, Type_
Variation location	13	cDNApos, relcDNApos, CDSpos, relCDSpos, protPos, relProtPos, minDistTSS, AnnoType_CodingTranscript, AnnoType_Intergenic, AnnoType_NonCodingTranscript, AnnoType_Transcript, Consequence_3PRIME_UTR, Consequence_5PRIME_UTR

TF Binding Motif	9	motifECount, motifEHIPos, motifEScoreChng, motifDist, minDistTSE, TFBS, TFBSPeaks, TFBSPeaksMax, tOverlapMotifs
Chromatin State	32	cHmm, Enc
Splicing	15	MMSp_acceptorIntron, MMSp_acceptor, MMSp_exon, MMSp_donor, MMSp_donorIntron, Consequence_SPLICE_SITE, Dst2Splice, SpliceAI-acc-gain, SpliceAI-acc-loss, SpliceAI-don-gain, SpliceAI-don-loss, Dst2SplType_ACCEPTOR, Dst2SplType_DONOR, Consequence_CANONICAL_SPLICE, dbscSNV-ada_score
GC Content	2	GC, CpG

[1] Missing values were generally replaced by 0. Missing values for the following features appear in parenthesis: Chromatin State features (0.067), SIFTval(1), GC(0.42), CqG(0.02), priPhCons(0.115), mamPhCons(0.079), verPhCons(0.094),priPhyloP(-0.033), mamPhyloP(-0.038), verPhyloP(0.017), GerpN(1.91), GerpS(-0.2).

Table S2. The dataset of variants.

Table S2A. Number of variants per tissue in the train dataset.

Tissue	Disease genes[1]	Pathogenic variants	Benign variants	Fraction of pathogenic variants	auROC	auPRC
Brain - Basal Ganglia	26	72	14471	0.005	0.86	0.03
Brain - Cerebellum	97	367	14176	0.025	0.96	0.42
Brain - Cortex	77	372	14171	0.026	0.93	0.30
Brain - Spinal Cord	32	143	14400	0.01	0.87	0.05
Heart	70	475	14068	0.033	0.93	0.47
Kidney	52	337	14206	0.023	0.93	0.38
Liver	30	106	14437	0.007	0.92	0.085
Lung	21	154	14389	0.01	0.87	0.11
Skeletal muscle	77	377	14166	0.026	0.95	0.49
Skin	53	279	14264	0.019	0.90	0.20
Testis	45	254	14289	0.017	0.79	0.085
Nerve	46	167	14376	0.011	0.90	0.20
Whole blood	31	111	14432	0.008	0.92	0.26
Whole brain	276	1083	13460	0.074	0.97	0.75
Median	49	266.5	14276.5	0.018	0.92	0.23

[1] Disease genes refer to genes that contain pathogenic variants.

Table S2B. Number of variants per tissue in the test dataset.

Tissue	Disease genes[1]	Pathogenic variants	Benign variants	Fraction of pathogenic variants	auROC	auPRC
Brain - Basal Ganglia	15	24	2405	0.01	0.93	0.65
Brain - Cerebellum	48	78	2351	0.03	0.99	0.90
Brain - Cortex	36	79	2350	0.033	0.996	0.95
Brain - Spinal Cord	15	30	2399	0.012	0.97	0.91
Heart	36	78	2351	0.032	0.97	0.91
Kidney	29	94	2335	0.039	0.99	0.91
Liver	16	26	2403	0.01	0.95	0.61
Lung	11	28	2401	0.012	0.97	0.90
Skeletal muscle	42	78	2351	0.03	0.98	0.88
Skin	27	51	2378	0.02	0.98	0.82
Testis	17	20	2409	0.008	0.95	0.58
Nerve	13	19	2410	0.008	0.97	0.75
Whole blood	22	35	2394	0.014	0.98	0.73
Whole brain	126	246	2183	0.10	0.99	0.94
Median	24.5	43	2386	0.018	0.98	0.89

[1] Disease genes refer to genes that contain pathogenic variants.

Table S2C. Number of variants per tissue in the limited test set (only variants in genes with no other variants in the training set were considered).

Tissue	Disease genes[1]	Pathogenic variants	Benign variants	Fraction of pathogenic variants	auROC	auPRC
Brain - Basal Ganglia	4	7	1527	0.005	0.86	0.02
Brain - Cerebellum	6	7	1527	0.005	0.95	0.24
Brain - Cortex	3	3	1531	0.002	0.98	0.075
Brain - Spinal Cord	0	0	1534	0		
Heart	3	4	1530	0.003	0.92	0.046
Kidney	6	8	1526	0.005	0.97	0.095
Liver	5	7	1527	0.005	0.89	0.102

Lung	2	2	1532	0.001	0.62	0.0016
Skeletal muscle	3	4	1530	0.0026	0.94	0.08
Skin	5	6	1528	0.004	0.96	0.3
Testis	2	3	1531	0.002	0.88	0.008
Nerve	3	3	1531	0.002	0.96	0.023
Whole blood	3	3	1531	0.002	0.93	0.35
Whole brain	15	20	1514	0.013	0.98	0.32
Median	3	4	1530	0.0026	0.94	0.08

[1] Disease genes refer to genes that contain pathogenic variants.

Table S2D. Number of variants per tissue in the full dataset.

Tissue	Disease genes[1]	Pathogenic variants	Benign variants	Fraction of pathogenic variants
Brain - Basal Ganglia	33	108	18526	0.006
Brain - Cerebellum	110	462	18172	0.025
Brain - Cortex	82	456	18178	0.0245
Brain - Spinal Cord	36	174	18460	0.0093
Heart	82	556	18078	0.0298
Kidney	61	434	18200	0.0233
Liver	40	142	18492	0.0076
Lung	25	184	18450	0.0099
Skeletal muscle	85	463	18171	0.0248
Skin	65	349	18285	0.01873
Testis	53	278	18356	0.0149
Nerve	51	191	18443	0.01
Whole blood	42	151	18483	0.008
Whole brain	311	1356	17278	0.073
Median	57	313.5	18320.5	0.0168

[1] Disease genes refer to genes that contain pathogenic variants.

Table S3. A summary of patient cases and the rank of the pathogenic variant by TRACEvar.

Table S3A. Patient cases metadata.

Case	Patient ID	Tissue	Disease Name	Number of Candidate Variants	Rank
AFOtB1305_Heart	OtB1305	Heart	Atrial fibrillation	528	263
AgenesisOfCC16550_Brain	16550	Brain	AGENESIS OF CORPUS CALLOSUM	111	56
Ataxia13863_Brain	13863	Brain	Ataxia	125	11
AtaxiaOtB0926_Brain	OtB0926	Brain	Ataxia	145	15
ATLDOt4998_Brain	Ot4998	Brain	Ataxia telangiectasia like disorder - ATLD	1499	165
Autism13498_Brain	13498	Brain	Autism	241	2
AzoospermiaPMRRP 28085_Brain	28085	Brain	Azoospermia + PMR + RP	151	3
AzoospermiaPMRRP 28085_Testis	28085	Testis	Azoospermia + PMR + RP	151	29
CardiacNeuralSyndrome41621_Brain	41621	Brain	Cardiac Neural Syndrome	114	5
CardiacNeuralSyndrome41621_Heart	41621	Heart	Cardiac Neural Syndrome	114	52
CardiacNeuralsyndromeOtA46052_Brain	OtA4605	Brain	PMR+Hypotonia	315	8
CardiomyopathyOtB0551_Heart	OtB0551	Heart	Cardiomyopathy	180	36
ComplexVDefOB13_Brain	OB13	Brain	Complex V deficiency	286	97
ComplexVDefOB13_ Skeletal muscle	OB13	Skeletal muscle	Complex V deficiency	286	123
DevDelay5148_Brain	5148	Brain	Developmental delay	181	22
diaphragmaticherniaOt2822_ Skeletal muscle	Ot2822	Skeletal muscle	diaphragmatic hernia	331	36
EctodermalOtB0548_Skin	OtB0548	Skin	Ectodermal dysplasia	515	3
EctodermalOtB0549_Skin	OtB0549	Skin	Ectodermal dysplasia	507	5
EctodermalOtB0550_Skin	OtB0550	Skin	Ectodermal dysplasia	544	4
HSP13393_ Skeletal muscle	13393	Skeletal muscle	Hereditary Spastic Paraparesis (HSP)	147	13

HSP16390_ Skeletal muscle	16390	Skeletal muscle	HSP	137	45
HSP5199_Brain	5199	Brain	Int disability+epilepsy +spastic q-plegia	680	256
HypotoniadevdelayOtA8152_Brain	OtA8152	Brain	hypotonia and global neuro-developmental delay	181	15
HypotoniadevdelayOtA8152_ Skeletal muscle	OtA8152	Skeletal muscle	hypotonia and global neuro-developmental delay	181	1
HypotoniaOt2812_ Skeletal muscle	Ot2812	Skeletal muscle	Hypotonia	1700	1158
Leighsyndrome766M_Brain	766M	Brain	Leigh syndrome	161	5
Metabolic16028_Brain	16028	Brain	Metabolic-Neurologic disease	770	205
Microcephaly2922_Brain	2922	Brain	microcephaly and epilepsy	199	34
MicrocephalyOB2_Brain	OB2	Brain	Microcephaly + PMR	326	13
MicrocephalyOB3_Brain	OB3	Brain	Microcephaly + PMR	286	6
MicrocephalyOB43_Brain	OB43	Brain	microcephaly	256	14
MicrocephalyOt1097_Brain	Ot1097	Brain	Microcephaly	1044	38
MicrocephalyOt5010_Brain	Ot5010	Brain	microcephaly	354	63
MicrocephalyOtB0918_Brain	OtB0918	Brain	Microcephaly + PMR	164	1
Migrane13859_Brain	13859	Brain	Complicated migraine	115	1
Moyamoya13700_Brain	13700	Brain	Moyamoya	169	35
Musculardystrophy13879_ Skeletal muscle	13879	Skeletal muscle	Muscular dystrophy	185	9
Myopathy13786_ Skeletal muscle	13786	Skeletal muscle	Myopathy	124	1
NPHPOB10_Brain	OB10	Brain	NPHP	270	48
NPHPOt1114_Brain	Ot1114	Brain	NPHP	321	52
NPHPOt1114_Kidney	Ot1114	Kidney	NPHP	321	117
NTDOtA4611_Brain	OtA4611	Brain	NTD	787	55
OpticAtrophy16012_Brain	16012	Brain	Optic atrophy plus	556	52
PCCAOt1103_Brain	Ot1103	Brain	PCCA	338	22

PCCAOt1104_Brain	Ot1104	Brain	PCCA	317	20
PMR1055M_Brain	1055M	Brain	Severe Retardation	129	7
PMR13595_Brain	13595	Brain	PMR	824	52
PMR16009_Brain	16009	Brain	Mental retardation syndrome	851	31
PMROt2823_Brain	Ot2823	Brain	Mental Retardation	328	34
SeckelSyndromeSS2_Brain	S2	Brain	Seckel Syndrome	181	43
Skin16264_Skin	16264	Skin	Ectodermal Dysplasia	102	54
SpasticParaparesisOt5005_ Skeletal muscle	Ot5005	Skeletal muscle	Hereditary Spastic Paraparesis (HSP)	1030	70
Strabismus13556_Brain	13556	Brain	strabismus, mental retardation, short stature	108	7
Strabismus13566_Brain	13566	Brain	strabismus, mental retardation, short stature	119	19
Usher16032_Brain	16032	Brain	Usher syndrome	172	1
Zellweger16114_Brain	16114	Brain	Zellweger syndrome	129	6
Zellwegersyndrome13414_Brain	13414	Brain	Zellweger syndrome	218	4

Table S3B. Description of Pathogenic variants.

Case	ACMG[1] Classification	Gene Name	Gene ID	Chromosome	Position	Reference	Alternative	Type
AFOtB1305_Heart	PM2 PP3 PS3 PP1	KCND2	ENSG00000184408	7	120381650	C	G	SNV
AgenesisOfCC16550_Brain	PM2 PP3 PP1	L1CAM	ENSG00000198910	X	153135948	C	T	SNV
Ataxia13863_Brain	PM2 PP3 PVS1 PP1	SACS	ENSG00000151835	13	23928836	ACA	A	DEL
AtaxiaOtB0926_Brain	PM2 PP3 PP2 PP1	ATM	ENSG00000149311	11	108121706	T	C	SNV
ATLDOt4998_Brain	PM2 PP3 PP1	MRE11A	ENSG00000020922	11	94219114	T	C	SNV
Autism13498_Brain	PM2 PP3 PS3 PP1	PAK3	ENSG00000077264	X	110385360	C	G	SNV
AzoospermiaPMRRP28085_Brain	PM2 PP3 PM4 PS3 PP1	SCAPER	ENSG00000140386	15	76866531	AG	A	DEL
AzoospermiaPMRRP28085_Testis	PM2 PP3 PM4 PS3 PP1	SCAPER	ENSG00000140386	15	76866531	AG	A	DEL
CardiacNeuralSyndrome41621_Brain	PM2 PP3 PM4 PP1	RECQL4	ENSG00000160957	8	145738492	AAT	A	DEL

CardiacNeuralSyndrome 41621_Heart	PM2 PP3 PM4 PP1	RECQL4	ENSG00000160957	8	145738492	AAT	A	DEL
CardiacNeuralsyndrome OtA46052_ Brain	PM2 PP3 PM4 PS3 PP1	RSRC1	ENSG00000174891	3	157841665	C	T	SNV
CardiomyopathyOtB0551_Heart	PM2 PP3 PS3 PP1	PLEKHM2	ENSG00000116786	1	16055171	AAG	A	DEL
ComplexVDefOB13_Brain	PM2 PP3 PP2 PP1	SLC25A1	ENSG00000100075	22	19164125	T	C	SNV
ComplexVDefOB13_ Skeletal muscle	PM2 PP3 PP2 PP1	SLC25A1	ENSG00000100075	22	19164125	T	C	SNV
DevDelay5148_brain	PM2 PP3 PP1 PP5	TBCD	ENSG00000141556	17	80828204	G	A	SNV
diaphragmatichernia Ot2822_ Skeletal muscle	PM2 PP3 PP1	SYNM	ENSG00000182253	15	99670425	G	T	SNV
Ectodermal OtB0548_Skin	PM2 PP3 PP1 PP5	TP63	ENSG00000073282	3	189584501	G	A	SNV
Ectodermal OtB0549_Skin	PM2 PP3 PP1 PP5	TP63	ENSG00000073282	3	189584501	G	A	SNV
Ectodermal OtB0550_Skin	PM2 PP3 PP1 PP5	TP63	ENSG00000073282	3	189584501	G	A	SNV
HSP13393_ Skeletal muscle	PM2 PP3 PM4 PS3 PP1	KY	ENSG00000174611	3	134369751	A	AATGTCGATAGATACAGCACATGTCGATA	INS
HSP16390_ Skeletal muscle	PM2 PP3 PP1	DSTYK	ENSG00000133059	1	205130494	G	A	SNV
HSP5199_Brain	PM2 PP3 PS3 PP1	DEGS1	ENSG00000143753	1	224377960	A	G	SNV
HypotoniadevdelayOtA8152_Brain	PM2 PP3 PM4 PS3 PP1	PAX7	ENSG00000009709	1	19071306	A	G	SNV
HypotoniadevdelayOtA8152_ Skeletal muscle	PM2 PP3 PM4 PS3 PP1	PAX7	ENSG00000009709	1	19071306	A	G	SNV
HypotoniaOt2812_ Skeletal muscle	PM2 PP3 PS3 PP1	CCDC174	ENSG00000154781	3	14712701	A	G	SNV

Leighsyndrome766M_Brain	PM2 PS1 PM1 PP1 PP5	COX15	ENSG00000014919	10	101483814	G	A	SNV
Metabolic16028_Brain	PM2 PP3 PVS1 PP1	TMEM70	ENSG00000175606	8	74888621	A	AT	INS
Microcephaly2922_Brain	PM2 PP3 PVS1 PP1	AIMP2	ENSG00000106305	7	6063012	ATC	A	DEL
MicrocephalyOB2_Brain	PM2 PP3 PM4 PS3 PP1	UNC80	ENSG00000144406	2	210640622	C	T	SNV
MicrocephalyOB3_Brain	PM2 PP3 PM4 PS3 PP1	UNC80	ENSG00000144406	2	210640622	C	T	SNV
MicrocephalyOB43_Brain	PM2 PP3 PP2 PP1	SBF1	ENSG00000100241	22	50902962	C	T	SNV
MicrocephalyOt1097_Brain	PM2 PP3 PS3 PP1	WDFY3	ENSG00000163625	4	85636503	G	A	SNV
MicrocephalyOt5010_Brain	PM2 PP3 PP1	STIL	ENSG00000123473	1	47716848	AA	A	DEL
MicrocephalyOtB0918_Brain	PM2 PP3 PM4 PS3 PP1	UNC80	ENSG00000144406	2	210640622	C	T	SNV
Migrane13859_Brain	PM2 PP3 PP1	CACNA1A	ENSG00000141837	19	13414691	G	A	SNV
Moyamoya13700_Brain	PM2 PP3 PP2 PP1 PP5	RNF213	ENSG00000173821	17	78343601	T	A	SNV
Musculardystrophy13879_ Skeletal muscle	PM2 PS1 PS3 PP1 PP5	MPV17	ENSG00000115204	2	27535925	C	T	SNV
Myopathy13786_ Skeletal muscle	PM2 PP3 PVS1 PP1	SGCG	ENSG00000102683	13	23898506	G	A	SNV
NPHPOB10_Brain	PM2 PP3 PS3 PP1	SLC30A9	ENSG00000014824	4	42067341	AAGC	A	DEL
NPHPOt1114_Brain	PM2 PP3 PS3 PP1	SLC30A9	ENSG00000014824	4	42067341	AAGC	A	DEL
NPHPOt1114_Kidney	PM2 PP3 PS3 PP1	SLC30A9	ENSG00000014824	4	42067341	AAGC	A	DEL
NTDOtA4611_Brain	PM2 PP3 PVS1 PP1 PP5	FKTN	ENSG00000106692	9	108382330	A	AA	INS
OpticAtrophy16012_Brain	PM2 PP3 PM1 PP1	OPA1	ENSG00000198836	3	193360578	C	G	SNV
PCCAOt1103_Brain	PM2 PP3 PS3 PP1	VPS53	ENSG00000141252	17	465738	C	T	SNV
PCCAOt1104_Brain	PM2 PP3 PS3 PP1	VPS53	ENSG00000141252	17	465738	C	T	SNV
PMR1055M_Brain	PM2 PP3 PM4 PS3 PP1	SEC31A	ENSG00000138674	4	83763483	A	ATA	INS
PMR13595_Brain	PM2 PP3 PM1 PP1	PGAP2	ENSG00000148985	11	3846278	G	A	SNV

PMR16009_ Brain	PM2 PP3 PP2 PP1 PP5	ZC4H2	ENSG00000126970	X	64137701	G	A	SNV
PMROt2823 _Brain	PM2 PP3 PM4 PP1	UBE3B	ENSG00000151148	12	109928849	G	A	SNV
SeckelSyndr omeSS2_Br ain	PM2 PP3 PVS1 PS3 PP1	CENPJ	ENSG00000151849	13	25459823	T	C	SNV
Skin16264_ Skin	PM2 PP3 PVS1 PP1	EOGT	ENSG00000163378	3	69037455	AT	A	DEL
SpasticParap aresisOt500 5_ Skeletal muscle	PM2 PP3 PM4 PS3 PP1	KY	ENSG00000174611	3	134369751	ACGAT CAGCA G	A	DEL
Strabismus1 3556_Brain	PM2 PP3 PP2 PP1	NF1	ENSG00000196712	17	29559867	C	A	SNV
Strabismus1 3566_Brain	PM2 PP3 PP2 PP1	NF1	ENSG00000196712	17	29559867	C	A	SNV
Usher16032 _Brain	PM2 PP3 PVS1 PP1	GPR98	ENSG00000164199	5	90059126	AT	A	DEL
Zellweger16 114_Brain	PM2 PP3 PVS1 PP1	PEX6	ENSG00000124587	6	42934534	AC	A	DEL
Zellwegersy ndrome1341 4_Brain	PM2 PP3 PM1 PP1	HSD17B4	ENSG00000133835	5	118814660	A	AT	INS

[1] The American College of Medical Genetics and Genomics (ACMG).

Table S4. Tissue-specific features used by the TRACEvar multi-tissue model.

Feature Group	Number of Features
Adult Expression	1
Preferential Expression	1
Process Activity	1
PPIs	1
Paralog Relationships	1
