# Supplementary material for: Tissue-aware interpretation of genetic variants advances the etiology of rare diseases
Source: Mol Syst Biol. 2024 Sep 16;20(11):4. doi: 10.1038/s44320-024-00061-6 (PMC11535248; doi:10.1038/s44320-024-00061-6)
Supplement: Supplementary file 6 — Expanded View Figures [file 44320_2024_61_MOESM6_ESM.pdf]

Expanded View Figures

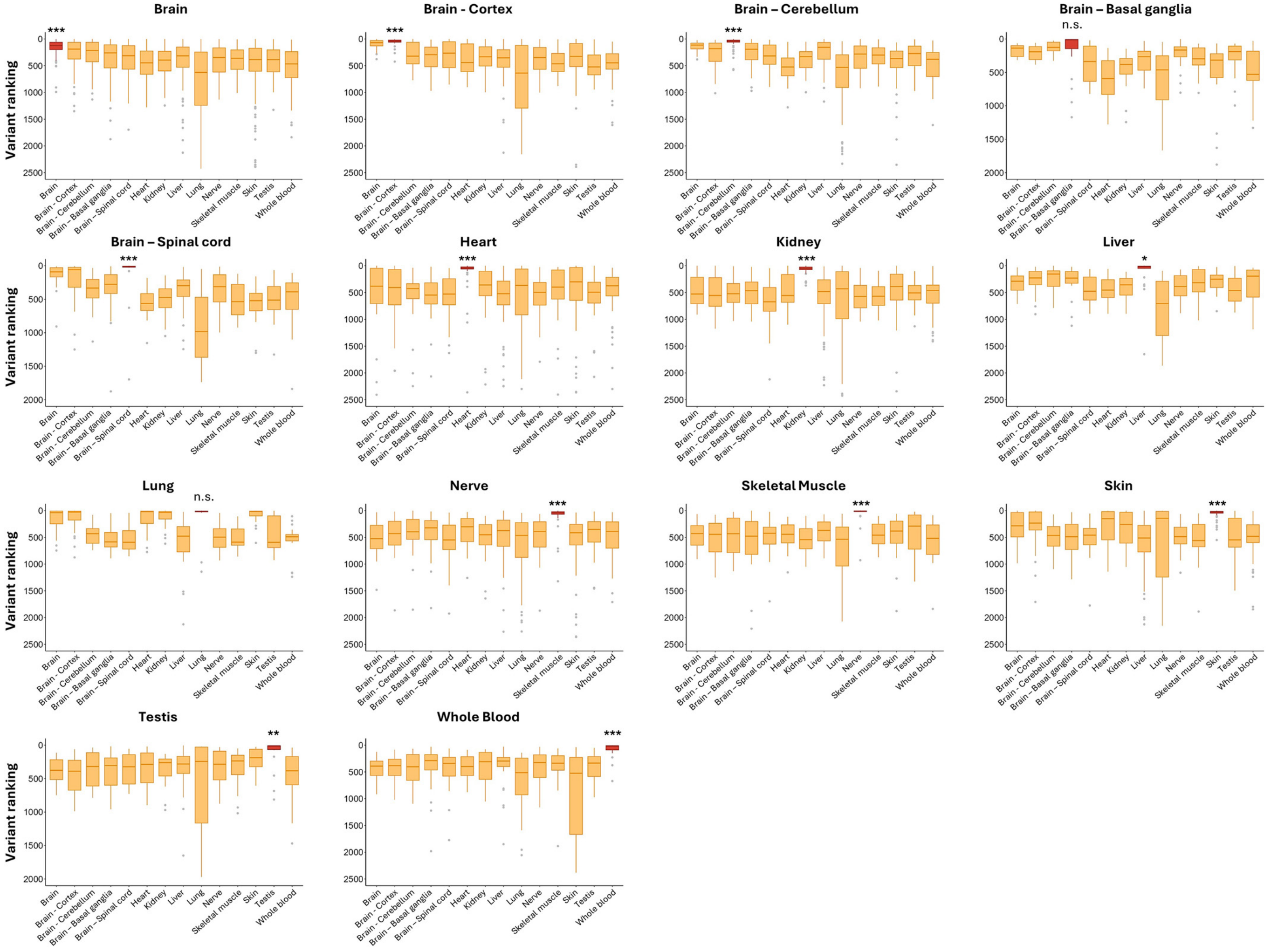

Figure EV1. Using TRACEvar to predict the tissue that is likely affected by a pathogenic variant.

Pathogenic variants of an affected tissue ($19 \le n \le 247$) were ranked by the model of the affected tissue (red) and by each of the other tissues. In all 14 tissues, the median rank of pathogenic variants was highest when ranked by the model of their affected tissue. One-tailed paired Wilcoxon test, adjusted P values: $*P < 0.05$, $**P < 0.01$, $***P < 0.001$, n.s. not significant. Boxplot central band indicates median; box limits indicate 25th to 75th percentiles; whiskers indicate 1.5 × interquartile range.

 © The Author(s)

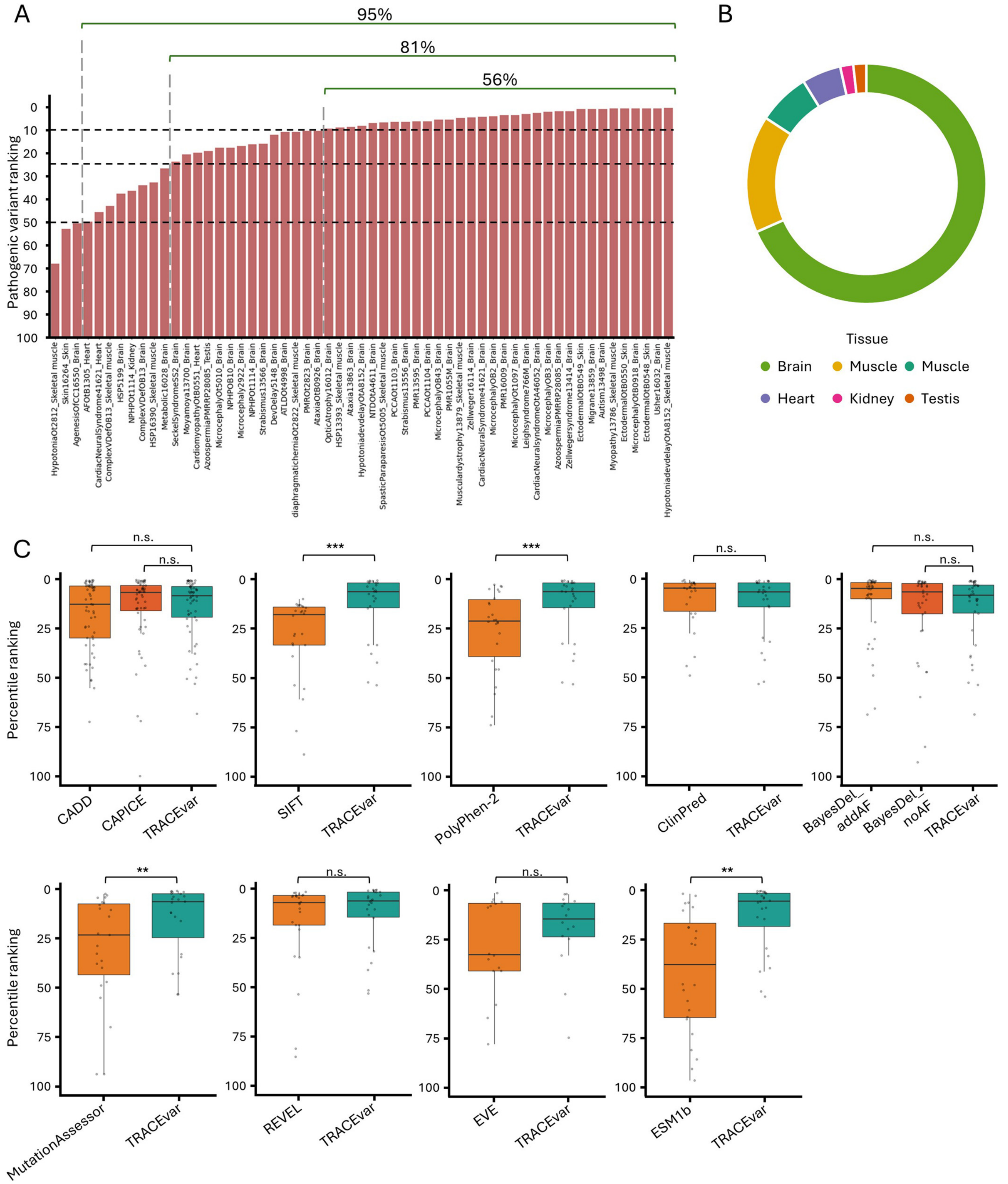
A
95%
81%
56%
Pathogenic variant ranking
0
10
20
30
40
50
60
70
80
90
100
HypotoniaOt2812_Skeletal muscle
Skin16264_Skin
AgenesisOfCC16550_Brain
AFOt81305_Heart
CardiacNeuralSyndrome41621_Heart
ComplexVDefOB13_Skeletal muscle
HSP5199_Brain
NPHPOt1114_Kidney
ComplexVDefOB13_Brain
HSP16390_Skeletal muscle
Metabolic16028_Brain
SeckelSyndrome552_Brain
Moyamoya13700_Brain
CardiomyopathyOtB0551_Heart
AzoospermiaPMRRP28085_Testis
MicrocephalyOt5010_Brain
NPHPOB10_Brain
Microcephaly2922_Brain
NPHPOt1114_Brain
Strabismus13566_Brain
DevDelay5148_Brain
ATLDOt4998_Brain
diaphragmaticherniaOt2822_Skeletal muscle
PMROt2823_Brain
AtaxiaOtB0926_Brain
OpticAtrophy16012_Brain
HSP13393_Skeletal muscle
Ataxia13863_Brain
HypotoniadevdelayOtA8152_Brain
NTDOtA4611_Brain
SpasticParaparesisOt5005_Skeletal muscle
PCCAOt1103_Brain
Strabismus13556_Brain
PMR13595_Brain
PCCAOt1104_Brain
MicrocephalyOB43_Brain
PMR1055M_Brain
Musculardystrophy13879_Skeletal muscle
Zellweger16114_Brain
CardiacNeuralSyndrome41621_Brain
MicrocephalyOB2_Brain
PMR16009_Brain
MicrocephalyOt1097_Brain
Leighsyndrome766M_Brain
CardiacNeuralsyndromeOtA46052_Brain
MicrocephalyOB3_Brain
AzoospermiaPMRRP28085_Brain
Zellwegersyndrome13414_Brain
EctodermalOtB0549_Skin
Migrane13859_Brain
Autism13498_Brain
Myopathy13786_Skeletal muscle
EctodermalOtB0550_Skin
MicrocephalyOtB0918_Brain
EctodermalOtB0548_Skin
Usher16032_Brain
HypotoniadevdelayOtA8152_Skeletal muscle
B
Tissue
Brain
Muscle
Muscle
Heart
Kidney
Testis
C
Percentile ranking
0
25
50
75
100
n.s.
n.s.
CADD
CAPICE
TRACEvar
***
SIFT
TRACEvar
***
PolyPhen-2
TRACEvar
n.s.
ClinPred
TRACEvar
n.s.
n.s.
BayesDel_addAF
BayesDel_noAF
TRACEvar
**
MutationAssessor
TRACEvar
n.s.
REVEL
TRACEvar
n.s.
EVE
TRACEvar
**
ESM1b
TRACEvar

◀ **Figure EV2. Assessment of TRACEvar performance on clinical cases.**

(**A**) The rank of the verified pathogenic variant out of the patient's candidate variants. 95% of the verified pathogenic variant ranked above the median; 81% ranked at the top quartile; 56% ranked at the top 10%, and 40% ranked at the top 5% of the variants per patient. (**B**) The distribution of cases per tissue. (**C**) Comparison between ranking of the verified pathogenic variant by TRACEvar and by other tools. TRACEvar performed significantly better than 4/10 tools, and similarly to most of the remaining tools. One-tailed paired Wilcoxon test, adjusted *P* values: $^{**}P < 0.01$, $^{***}P < 0.001$, n.s. not significant. The exact *P* values appear in the Source Data.

© The Author(s)
